# Epidemiological and clinical characteristics of psittacosis among cases with complicated or atypical pulmonary infection using metagenomic next-generation sequencing: a multi-center observational study in China

**DOI:** 10.1186/s12941-023-00631-w

**Published:** 2023-09-07

**Authors:** Weifeng Huang, Fengge Wang, Qingqing Cai, Huiliang Xu, Dengwei Hong, Han Wu, Lu Zhou, Linjie Hu, Yihan Lu

**Affiliations:** 1 Department of Intensive Care Medicine, The Sixth People’s Hospital, Shanghai Jiao Tong University School of Medicine, Shanghai, China; 2 Shanghai Institute of Infectious Disease and Biosecurity, Fudan University, Shanghai, China; 3 Genoxor Medical Science and Technology Inc, Shanghai, China; 4 Department of Respiratory, Nanxiang Hospital of Jiading district, Shanghai, China; 5 Department of Epidemiology, Ministry of Education Key Laboratory of Public Health Safety, Fudan University School of Public Health, Shanghai, China

**Keywords:** Psittacosis, Parrot fever, Metagenomic next-generation sequencing, Pulmonary infection, Epidemiology

## Abstract

**Background:**

*Chlamydia psittaci* (*C. psittaci*) causes parrot fever in humans. Development of metagenomic next-generation sequencing (mNGS) enables the identification of *C. psittaci*.

**Methods:**

This study aimed to determine the epidemiological and clinical characteristics of parrot fever cases in China. A multi-center observational study was conducted in 44 tertiary and secondary hospitals across 14 provinces and municipalities between April 2019 and October 2021.

**Results:**

A total of 4545 patients with complicated or atypical pulmonary infection were included in the study, among which the prevalence of *C. psittaci* was determined to be 2.1% using mNGS. The prevalence of *C. psittaci* was further determined across demographic groups and types of specimens. It was significantly higher in patients with senior age (2.6% in those > 50 years), winter-spring (3.6%; particularly in December, January, and February), and southwestern (3.4%) and central and southern China (2.7%) (each P < 0.001). Moreover, the prevalence was the highest in bronchoalveolar lavage fluid (BALF) (2.9%), compared with sputum (1.1%) and peripheral blood specimens (0.9%). Additionally, co-infection of principal microorganisms was compared. Certain microorganisms were more likely to co-infect in parrot fever cases, such as *Candida albicans* in BALF (26.7%) and peripheral blood (6.3%), compared with non-parrot fever cases (19.7% and 1.3%); however, they did not significantly differ (each *P* > 0.05).

**Conclusion:**

Parrot fever remains low in patients with complicated or atypical pulmonary infection. It is likely to occur in winter-spring and southwestern region in China. BALF may be the optimal specimen in the application of mNGS. Co-infection of multiple microorganisms should be further considered.

## Background

Psittacosis, also known as parrot fever and ornithosis, is a zoonotic infectious disease caused by *Chlamydia psittaci* (*C. psittaci*) that is aerobic gram-negative bacterium [1]. Birds are natural hosts of *C. psittaci* that could spread through feces and respiratory secretions [2]. Humans become infected with *C. psittaci* through direct contact with infected birds or by inhaling aerosols or dust containing *C. psittaci* [3]. Furthermore, human-to-human transmission of *C. psittaci* has been documented [1, 4]. All birds and humans of all ages are susceptible to *C. psittaci*; however, it is more common in adults, especially those who have contact with pet birds and poultry including pet bird owners, pet store employees, poultry slaughter workers, and veterinarians [5]. Parrot fever is sporadic worldwide, such as the USA [6], Europe [7, 8], Australia [9], and Japan [10]. In China, multiple cases of parrot fever have been increasingly reported in recent years [11, 12], suggesting it remains a public health concern.

Parrot fever mainly attacks the lungs and may subsequently cause systemic disease. After inhalation through the respiratory tract, *C. psittaci* proliferates in the local mononuclear macrophage system, then enters the blood and spreads to the lungs and other organs through blood circulation. Atypical pneumonia is the most common manifestation, with fever, chills, headache, myalgia, cough, and pulmonary infiltration, which are similar to other respiratory diseases [1, 13]. Parrot fever causes a wide range of illness and severity, from asymptomatic to life-threatening. Most patients develop mild symptoms and have good clinical outcomes [9]. However, in rare cases, they might have severe pneumonia and other organ dysfunction [1]. The mortality has been documented to be 1% in elderly [14]. Moreover, parrot fever has been estimated to contribute approximately 1.03% of community-acquired pneumonia (CAP) [13]. It is crucial in the clinical diagnosis of parrot fever.

However, it remains a challenge in the diagnosis of parrot fever due to following reasons. First, the public and physicians have limited knowledge and awareness of parrot fever and usually do not consider the diagnosis of *C. psittaci* infection. Second, clinical manifestation of parrot fever is similar to other respiratory diseases, which might lead to underdiagnosis or misdiagnosis [14, 15]. Third, routine diagnostic methods, including culture, serological testing, and Polymerase chain reaction (PCR), have limited accuracy or timeliness of the diagnosis. Traditional culture of *C. psittaci* may take long time and be usually negative. Serological testing may be easily interfered by cross reaction, which is often considered as a supportive test instead of optimal diagnostic method [16]. PCR is a targeted test and would be utilized with assumption about pathogens [17]. In recent years, metagenomic next-generation sequencing (mNGS) has been developed as a non-targeted testing method, which does not require specific amplification and could identify rare pathogens, making it possible to diagnose atypical pathogens with advantages of high throughput, high sensitivity and rapid detection [18]. Up till now, multiple studies have highlighted its value and advantages in pathogen detection compared with routine methods [19–21]. Consequently, parrot fever cases are increasingly reported by using mNGS in China. However, current clinical studies have been dominated by case reports of parrot fever, and provided insufficient epidemiology of parrot fever [22]. Therefore, this study aimed to determine the epidemiological and clinical characteristics of parrot fever among the patients with complicated or atypical pulmonary infection across 14 provinces and municipalities in China.

## Materials and methods

### Study design

We designed a multi-center observational study on hospitalized patients in 44 tertiary and secondary hospitals in 14 provinces and municipalities of China between April 2019 and October 2021. Inclusion criteria of patients were presented as follows: (1) symptoms of recent cough/sputum or original respiratory diseases were aggravated, with or without purulent sputum/chest pain/dyspnea/hemoptysis; (2) fever > 37.4 °C; (3) lung consolidation and/or wet rale; (4) peripheral blood leukocytes > 10 × 10^9^/L or < 4 × 10^9^/L, with or without left nuclear shift; and (5) chest imaging examination showed new patchy infiltration, lobar/segmental consolidation, ground glass opacity, or interstitial changes, with or without pleural effusion. Exclusion criteria were as follows: (1) patients who had underlying chronic respiratory diseases, such as asthma; (2) those who had liver and kidney dysfunction, hematological diseases, or autoimmune diseases; or (3) those whose clinical records were unavailable.

Patients’ data were collected, including their diagnosis time, types of specimens, findings in the mNGS examination (if tested positive for *C. psittaci*, number of DNA sequence reads were collected), in addition to demographics such as sex, age, and region, in the hospital information system and laboratory information system.

### Specimen collection

The patients with pneumonia or pulmonary infection received the mNGS examination within 5 days following their medical visits. Generally, they provided 3mL of bronchoalveolar lavage fluid (BALF) and/or sputum for mNGS examination. Some of the patients also provided other respiratory specimens (such as oral and nasolaryngeal secretion, pleural fluid, and lung tissue) when BALF and sputum was not available, and other specimens (including peripheral blood and cerebrospinal fluid) when physicians considered potential infection in other sites. All the specimens were collected and stored applying the principle of aseptic operation to avoid contamination. Peripheral blood specimens were stored in the cell-free DNA storage tube at room temperature, and other body fluid specimens were stored at 4 °C [23].

### Metagenomic sequencing and data preprocessing

First, DNA and RNA were extracted using Magnetic serum/plasma DNA Maxi kit (Tiangen Biotech (Beijing) Co. Ltd., China). For BALF specimens, HostZERQ Microbial DNA kit (Jianshi Biotech (Beijing) Co. Ltd., China) was used to remove human nucleic acids for further nucleic acid extraction. Second, nucleic acids were fragmented into 150–300 bp in length with Bioruptor (Diagenode Diagnostics, Belgium) that is a non-contact ultrasonic disruptor. The library was constructed using Library Preparation kit (Kapabio System, Boston, MA). Third, high-throughput sequencing was conducted with Illumina Next Seq550Dx system (Illumina Inc., San Diego, CA). In the process of sequencing, adaptors, reads with low quality and repeated sequences, and short reads < 36 bp in length were removed. Microorganisms were then identified in the specimens through sequence alignment in the microbial genome database (bacteria, viruses, fungi and parasites) by using Bowtie2 (version 2.3.5) (Genoxor Medical Science and Technology Inc., Shanghai) [24].

### Measurement

In this study, prevalence of *C. psittaci* was calculated in the cases with complicated or atypical pulmonary infection, in total and stratified by age (0–18, 19–30, 31–50, and > 50), calendar months, geographical regions (southwestern, eastern, central and southern, and northern), and types of specimens (BALF, sputum, peripheral blood, and others). In addition, this study compared the prevalence of other principal pathogens between parrot fever cases and non-parrot fever cases.

### Statistical analysis

Age was presented using both mean ± standard deviation (SD) and groups. Other categorical variables were described using proportions. R 4.1.1 (Foundation for Statistical Computing, Vienna, Austria) was used to plot. Student t test, chi-square test, and Fisher’s exact test were employed to compare the characteristics between the parrot fever cases and non-parrot fever cases when applicable. Pearson correlation was conducted to determine the correlation in the sequence reads of *C. psittaci* between various types of specimens in same cases. SPSS 25.0 (IBM, Armonk, NY) was utilized for statistical analysis in this study. A *P* value of < 0.05 was considered statistically significant.

## Results

### Prevalence of parrot fever

From April 2019 to October 2021, a total of 4545 patients diagnosed with pneumonia or pulmonary infection were included in the study. Of them, 96 (2.1%) tested positive for *C. psittaci*. Moreover, among the parrot fever cases, 58 (60.4%) developed severe pneumonia and 17 had complications of fever, multiple organ failure/dysfunction, or respiratory failure. In contrast, 581 cases (1.3%) among non-parrot fever cases had severe pneumonia, which was significantly lower to parrot fever cases (*P* < 0.001).

### Epidemiological characteristics

Prevalence of parrot fever differed by sex and age. *C. psittaci* was detected in 1.8% (52/2908) of male cases, whereas 2.7% (44/1637) of female cases (*P* = 0.043) (Table 1). Average age of parrot fever cases (61.8 ± 12.6 year) was significantly higher than that of non-parrot fever cases (54.9 ± 22.2 year) (*P* = 0.003). Furthermore, the prevalence significantly differed across age groups: 0 in children or adolescents ≤ 18 years, 0.4% in 19–30 years (1/253), 1.8% in 31–50 years (14/797), and 2.6% in those > 50 years (81/3079) (*P* < 0.001) (Table 1). Notably, stratified by age < 60 and ≥ 60 years, male and female cases were similar in age (each *P* > 0.05).

**Fig. 1:**
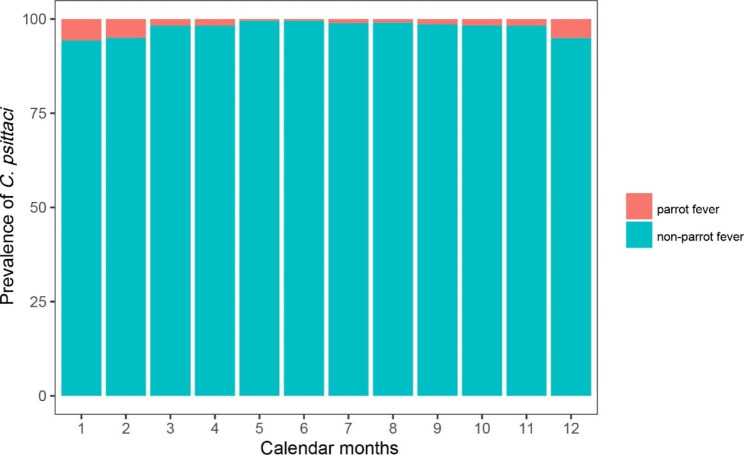
Examination of C. psittaci in the patients with complicated or atypical pulmonary infection by calendar months between April 2019 and October 2021

Moreover, prevalence of parrot fever showed the seasonality. It peaked in December, January, and February (all > 5%), while was lowest during May-June (0.5%) (Fig. 1). Furthermore, it was significantly different between winter-spring (November through April) (3.6%) and summer-autumn (May through October) (1.1%) (*P* < 0.001) (Table 1).

Additionally, spatial prevalence of parrot fever differed significantly (*P* < 0.001) (Table 1). We classified four regions, among which the prevalence was the highest in southwestern China (3.4%, 50/1465), followed by central and southern China (2.7%, 21/780), eastern China (1.3%, 18/1384), and northern China (0.8%, 7/916).

**Table 1:** Epidemiological characteristics between parrot fever cases and non-parrot fever cases

	Parrot fever cases (%)	Non-parrot fever cases (%)	*P* value
Average age (SD), year	61.8 (12.6)	54.9 (22.2)	0.003
Age group, year			< 0.001
0–18	0	416 (100)	
19–30	1 (0.4)	252 (99.6)	
31–50	14 (1.8)	783 (98.2)	
> 50	81 (2.6)	2998 (97.4)	
Sex			0.043
Male	52 (1.8)	2856 (98.2)	
Female	44 (2.7)	1593 (97.3)	
Region			< 0.001
Southwestern	50 (3.4)	1415 (96.6)	
Central and southern	21 (2.7)	759 (97.3)	
Eastern	18 (1.3)	1366 (98.7)	
Northern	7 (0.8)	909 (99.2)	
Season^a^			< 0.001
Winter-spring	68 (3.6)	1824 (96.4)	
Summer-autumn	28 (1.1)	2625 (98.9)	
Specimen type			< 0.001
BALF	86 (2.9)	2834 (97.1)	
Sputum	8 (1.1)	726 (98.9)	
Peripheral blood	16 (0.9)	1789 (99.1)	
Others^b^	0	361 (100)	

^a^Winter-spring, from November through April; summer-autumn, from May through October.

^b^Throat swab (n = 34), pleural fluid (n = 124), oral and nasolaryngeal secretion (n = 93), and lung tissue (n = 110).

### Clinical characteristics

In this study, a total of 6241 specimens of various types were collected. Majority of specimens were BALF (n = 2920, 46.8%), sputum (n = 734, 11.8%), and peripheral blood specimens (n = 1805, 28.9%), among which the prevalence of *C. psittaci* was 2.9% (86/2920), 1.1% (8/734), and 0.9% (16/1805), respectively (Table 1). In contrast, no *C. psittaci* was detected in cerebrospinal fluid (n = 246), pleural fluid (n = 124), oral and nasolaryngeal secretion (n = 127), lung tissue (n = 110) and other samples.

In the specimens positive for *C. psittaci*, sequence reads identified by mNGS ranged between 3 and 435,355 reads in BALF, 1 to 222,847 reads in sputum, and 1 to 227 reads in peripheral blood specimens. Furthermore, BALF and peripheral blood specimens were collected simultaneously in 10 cases (r = 0.485, *P* = 0.155), and sputum and peripheral blood specimens were collected simultaneously in 4 patients (r=-0.366, *P* = 0.634), which showed no significant correlation in the reads of *C. psittaci* between various types of specimens in same cases.

Additionally, multiple microorganisms were identified using mNGS. Certain pathogens that may cause illness were selected for further analysis. These pathogens were likely to be tested in the non-parrot fever cases, except *Candida albicans* in BALF and peripheral blood specimens, Epstein-Barr virus and *Staphylococcus epidermidis* in peripheral blood specimens (Tables 2 and 3, and 4); however, they did not significantly differ between parrot fever cases and non-parrot fever cases (each *P* > 0.05). Furthermore, co-infection prevalence of *Streptococcus pneumonia* (27.9%) and *Candida albicans* (26.7%) was higher than other pathogens in BALF (each *P* > 0.05) in the parrot fever cases.

**Table 2:** Co-infection of principal pathogens in BALF

Pathogens	Parrot fever cases n = 86 (%)	Non-parrot fever cases n = 2834 (%)	*P* value
*Streptococcus pneumoniae*	24 (27.9)	972 (34.3)	0.218
*Candida albicans*	23 (26.7)	559 (19.7)	0.108
*Haemophilus parainfluenzae*	16 (18.6)	778 (27.5)	0.069
*Streptococcus pseudopneumoniae*	14 (16.3)	684 (24.1)	0.092
*Klebsiella pneumoniae*	12 (14.0)	435 (15.3)	0.723

**Table 3:** Co-infection of principal pathogens in sputum

Pathogens	Parrot fever cases n = 8 (%)	Non-parrot fever cases n = 726 (%)	*P* value
*Candida albicans*	3 (37.5)	281 (38.7)	1.000
*Streptococcus pneumoniae*	2 (25.0)	269 (37.1)	0.717
*Corynebacterium striatum*	2 (25.0)	202 (27.8)	1.000
Epstein-Barr virus	1 (12.5)	216 (29.8)	0.448
Human herpesvirus 1	1 (12.5)	200 (27.5)	0.457
*Haemophilus parainfluenzae*	1 (12.5)	163 (22.5)	0.692
*Klebsiella pneumoniae*	0	239 (32.8)	0.059
*Acinetobacter baumannii complex*	0	221 (30.4)	0.114
*Streptococcus pseudopneumoniae*	0	195 (26.9)	0.118
*Pseudomonas aeruginosa*	0	140 (19.3)	0.364
*Stenotrophomonas maltophilia*	0	139 (19.2)	0.364

**Table 4:** Co-infection of principal pathogens in peripheral blood specimens

Pathogens	Parrot fever cases n = 16 (%)	Non-parrot fever cases n = 1789 (%)	*P* value
Epstein-Barr virus	4 (25.0)	164 (9.2)	0.054
*Candida albicans*	1 (6.3)	23 (1.3)	0.194
*Staphylococcus epidermidis*	1 (6.3)	15 (0.8)	0.133
*Klebsiella pneumoniae*	0	110 (6.2)	0.619
Human herpesvirus 1	0	92 (5.1)	1.000
*Acinetobacter baumannii complex*	0	64 (3.6)	1.000
*Pseudomonas aeruginosa*	0	32 (1.8)	1.000
*Corynebacterium striatum*	0	25 (1.4)	1.000
*Streptococcus pneumoniae*	0	18 (1.0)	1.000
*Stenotrophomonas maltophilia*	0	18 (1.01)	1.000
*Haemophilus influenzae*	0	9 (0.50)	1.000

## Discussion

This study determined the prevalence of parrot fever to be 2.1% in patients with complicated or atypical pulmonary infection across 14 provinces and municipalities in China during 2019–2021. It has been documented that the prevalence of parrot fever remains low and sporadic, which is 0-2.1% as described elsewhere [25]. The trend of parrot fever differs by countries and years. In the USA, a total of 935 parrot fever cases have been recorded in 1988–2003, whereas 112 cases in 2003–2014, showing a decline in the prevalence [1, 26]. In Belgium, number of parrot fever increased slowly since 2010, and in 2017, the number reported has almost doubled over the two previous years [27]. In China, increasing cases of parrot fever have been reported in 2009–2022, especially in Zhejiang, Hubei, and Sichuan provinces, suggesting a possibly increasing trend. Moreover, parrot fever differs by patient populations. In a meta-analysis, prevalence of parrot fever has been estimated based on the proportion of patients infected with *C. psittaci* in those CAP patients, which was 1.03% (95%CI, 0.79–1.30%) [13]. Our study targeted the patients with complicated or atypical pulmonary infection, in which possible pathogens might be difficult to confirm. Thus, we performed the mNGS to examine the prevalence of *C. psittaci*, which would provide evidence for better clarifying the prevalence of parrot fever including diagnosis and epidemiology in humans.

In previous studies, routine testing methods, including culture, complement fixation test, micro-immunofluorescence (MIF), and PCR, had been implemented. However, these methods have various limitations in the examination of *C. psittaci*, such as low sensitivity/specificity and long detection time, leading to underdiagnosed or misdiagnosed parrot fever [15, 17, 20]. Recently, mNGS has been widely applied in the identification of pathogens due to following advantages: (a) mNGS has higher sensitivity and specificity, particularly for atypical pathogens with low copies [19], compared with culture and serological testing. It results in a higher detection capability of possible pathogens [28]. (b) mNGS could identify uncommon and unknown pathogens, compared with targeted detection methods including serological testing and PCR. Moreover, it could determine potential co-infection of multiple pathogens such as in respiratory tract and central nervous system [19, 29]. (c) mNGS may achieve more rapid detection. In our study, average time between specimen collection and examination report of *C. psittaci* was 1.9 days, similar to 2–3 days described elsewhere [16]. Therefore, mNGS facilitated more rapid identification of *C. psittaci* and subsequently achieved accurate clinical diagnosis. Additionally, in traditional clinical practice, prevalence of *C. psittaci* might be underestimated due to use of antibiotics in advance [14, 30]. In our study, mNGS was conducted in the specimens collected within 2 days after admission, which might maximum the accuracy of detection though self-administration of antibiotics remained common before admission.

In this study, we determined the epidemiological characteristics of parrot fever among the patients with complicated or atypical pulmonary infection. Prevalence of parrot fever was significantly higher in patients with senior age (> 50 years), winter-spring (particularly in December, January, and February), and southwestern China and central and southern China. It may be associated with exposure to birds, such as direct contact with pet birds in pet stores and chicken, ducks, and pigeons in wet markets, which is more common in Chinese elderly than young generation [5, 31, 32]. Moreover, *C. psittaci* can survive for at least 72 h at 56 °C, several months in dry bird droppings, and even longer in freeze drying [33]. In our study, we observed highest prevalence of *C. psittaci* in December, January, and February, which is winter in China. However, parrot fever has been likely to occur in spring and summer in the Netherlands [34]. Another study in the Netherlands further showed that parrot fever occurred significantly higher in spring than that in other seasons [25]. The seasonality may be further studied. Additionally, multiple case reports found that parrot fever might be more common in southwestern China and southern China [12, 35]. Our findings provided similar evidence; however, it might be biased by the difference in the capacity and implementation of mNGS in clinical practice across regions in China. Therefore, it warrants further epidemiological investigation for more evidence.

Moreover, we explored the clinical characteristics of parrot fever. The majority (87.5%) of the parrot fever cases were admitted to the departments of respiratory medicine and intensive care medicine, suggesting they had severe infection. However, in all patients included in our study, complicated or atypical pulmonary infection differed by severity. Pulmonary infection is principal manifestation of parrot fever, so BALF may be recommended for examination. In our study, prevalence of *C. psittaci* was 2.9% in BALF and 1.1% in sputum, suggesting BALF may be the optimal specimen for examination of *C. psittaci*. Furthermore, peripheral blood specimens may be optional when respiratory specimens are not available in patients with pulmonary infection [15]. Cell-free DNA of *C. psittaci* in the cells can be released into peripheral blood after apoptosis in the lungs [36]. Previous study revealed that DNA copies of *C. psittaci* in BALF were significantly higher than in blood specimens [17]. In our study, we did not find the correlation in the reads of *C. psittaci* between various types of specimens in 14 cases with both peripheral blood and respiratory specimens. Number of reads is usually related to the collection time and sites of specimens, and possible interaction by co-infection [37]. Nevertheless, detection of *C. psittaci* in peripheral blood remains crucial for clinical diagnosis, regardless of the number.

Additionally, co-infection of multiple microorganisms was common among the patients with complicated or atypical pulmonary infection. We found *Candida albicans* was more likely to exist in BALF and peripheral blood specimens in parrot fever cases, while other principal pathogens were likely to be tested in non-parrot fever cases including *Streptococcus pneumoniae*, *Klebsiella pneumoniae*, *Haemophilus parainfluenzae*, *Staphylococcus epidermidis*, Human herpesvirus 1, and *Acinetobacter baumannii* complex. However, they did not significantly differ between parrot fever cases and non-parrot fever cases. It might be attributable to the fact that these pathogens are very common in pulmonary infection. Notably, we found a higher co-infection prevalence of Epstein-Barr virus and *Staphylococcus epidermidis* in peripheral blood specimens of parrot fever cases. Epstein-Barr virus is a human herpesvirus that causes systemic infection by infecting B lymphocytes [38]. S*taphylococcus epidermidis* is a normal bacterial community in the skin mucosa of the body, which might be detected due to contamination during sampling, instead of real blood infection. If multiple pathogens are detected in peripheral blood, it would indicate serious bloodstream infection. Thus, our findings proved parrot fever cases may have multiple co-infections of pathogens, which would exacerbate the disease. Clinical treatment should be tailored based on the co-infection of *C. psittaci* and other pathogens [39].

This study also had some limitations. First, we included the patients with complicated or atypical pulmonary infection across 14 provinces and municipalities in China. Limited study regions and implementation of mNGS might result in selection bias. Furthermore, complicated or atypical pulmonary infection might differ by severity across the departments and hospitals, due to variation in the judgment of disease severity. Second, we collected only respiratory or peripheral blood specimens in the parrot fever patients. It could not achieve the comparison between various types of specimens for examination of *C. psittaci*. In addition, we did not detect *C. psittaci* in pleural fluid, oral and nasolaryngeal secretion, lung tissue, or cerebrospinal fluid, which might underestimate the prevalence. Third, we included basic demographics of the patients, while did not collect more information such as other laboratory testing. Nevertheless, our study had the strength. Compared with case reports, this study illustrated a scenario of parrot fever epidemiology in China based on a 2.5-year observational study of a moderate sample size.

## Conclusion

Prevalence of parrot fever remains low and sporadic in China. It was significantly associated with senior age, winter-spring, and certain regions such as southwestern China and central and southern China. Application of mNGS showed an optimal performance for clinical diagnosis in the detection of *C. psittaci*, particularly in BALF. Moreover, parrot fever cases might have diverse co-infection of other principal pathogens, such as *Candida albicans*, Epstein-Barr virus, and *Staphylococcus epidermidis*. It warrants further studies on the influence of co-infection on the disease severity.

## Data Availability

The datasets used during the current study are available from the corresponding author on reasonable request.

## Abbreviations

*C. psittaci*: *Chlamydia psittaci*
mNGS: Metagenomic next-generation sequencing
BALF: Bronchoalveolar lavage fluid
CAP: Community-acquired pneumonia
PCR: Polymerase chain reaction
SD: Standard deviation
MIF: Micro-immunofluorescence
